# Altered Visual Function in Short-Wave-Sensitive 1 (*sws1*) Gene Knockout Japanese Medaka (*Oryzias latipes*) Larvae

**DOI:** 10.3390/cells12172157

**Published:** 2023-08-28

**Authors:** Ke Lu, Jiaqi Wu, Shulin Tang, Yuye Wang, Lixin Zhang, Farui Chai, Xu-Fang Liang

**Affiliations:** 1 College of Fisheries, Chinese Perch Research Center, Huazhong Agricultural University, Wuhan 430070, China; luke@webmail.hzau.edu.cn (K.L.); wangyuye@webmail.hzau.edu.cn (Y.W.);; 2 Engineering Research Center of Green Development for Conventional Aquatic Biological Industry in the Yangtze River Economic Belt, Ministry of Education, Wuhan 430070, China

**Keywords:** *sws1*, food intake, visual development, medaka larvae

## Abstract

Visual perception plays a crucial role in foraging, avoiding predators, mate selection, and communication. The regulation of color vision is largely dependent on opsin, which is the first step in the formation of the visual transduction cascade in photoreceptor cells. Short-wave-sensitive 1 (*sws1*) is a visual pigment that mediates short-wavelength light transduction in vertebrates. The depletion of *sws1* resulted in increased M-opsin in mice. However, there is still no report on the visual function of *sws1* in teleost fish. Here, we constructed the *sws1* knockout medaka using CRISPR/Cas9 technology. The 6 dph (days post-hatching) medaka *sws1*^−/−^ larvae exhibited significantly decreased food intake and total length at the first feeding stage, and the mRNA levels of orexigenic genes (*npy* and *agrp*) were significantly upregulated after feeding. The swimming speed was significantly reduced during the period of dark-light transition stimulation in the *sws1*-mutant larvae. Histological analysis showed that the thickness of the lens was reduced, whereas the thickness of the ganglion cell layer (GCL) was significantly increased in *sws1*^−/−^ medaka larvae. Additionally, the deletion of *sws1* decreased the mRNA levels of genes involved in phototransduction (*gnb3b*, *grk7a*, *grk7b,* and *pde6c*). We also observed increased retinal cell apoptosis and oxidative stress in *sws1* knockout medaka larvae. Collectively, these results suggest that *sws1* deficiency in medaka larvae may impair visual function and cause retinal cell apoptosis, which is associated with the downregulation of photoconduction expression and oxidative stress.

## 1. Introduction

The animal visual systems play vital roles in foraging, avoiding predators, mate selection, and communication [1]. In vertebrates, the retina is a multilayered tissue whose photoreceptor cells express opsin genes that are the core of vision at the molecular level [2]. Compared with other vertebrates, teleost fish have a unique set of visual opsins [3,4,5]. In fish, UV (short-wavelength sensitivity 1: *sws1*), blue (short-wavelength sensitivity 2: *sws2*), green (medium-wavelength sensitivity: *rh2*), and red (long-wavelength sensitivity: *lws*) opsins are expressed in cones and mediate color perception under strong light, and rod opsin (*rh1*) is expressed in rod cells and plays a role in dim light [6,7]. Some species have only a subset of these photopigment types, while others have multiple copies. Fish typically have fewer copies of short-wavelength-sensitive opsin (*sws1* and *sws2*) than long-wavelength-sensitive opsin genes, and *sws1* does not appear to have been ancestrally duplicated in teleost fish [8].

*sws1* belongs to the G-protein-coupled receptor family, and is highly expressed in planktonic fish owing to its ability to enhance ultraviolet light sensitivity [9,10]. To date, most fish studied have the SWS1 pigment, although many fish lose SWS1 (UV-sensitive) with maturity. This heterochronous expression may cause changes in spectral sensitivity, thereby adjusting the visual system of fish to adapt to environmental spectra [11,12]. For instance, African cichlid fishes that feed on zooplankton, algae, and phytoplankton display higher levels of *sws1* expression than those that feed on fish or benthic invertebrates [13]. In mice, the disruption of *sws1* resulted in the increased expression of M-opsin but did not cause retinal degeneration [14]. Recently, it was demonstrated that *sws1* deletion not only led to head and eye malformations but also retinal developmental defects in rainbow trout [15]. However, little is known about the relationship between *sws1* and visual dysfunction in fish larvae.

The teleost fish, medaka (*Oryzias latipes*) is an excellent model organism for the investigation of visual development and function [16,17]. Medaka has outstanding vision and still retains all four of the ancient pyramidal subtypes of vertebrates [18,19]. In the present study, we constructed *sws1* knockout medaka using CRISPR/Cas9 technology. We identified a *sws1* mutant zebrafish line with a 214 bp deletion, which resulted in a frameshift and premature stop codon. We further explored the visual function of *sws1* in larvae.

## 2. Results

### 2.1. Generation of sws1 Knockout Medaka

**Figure 1:**
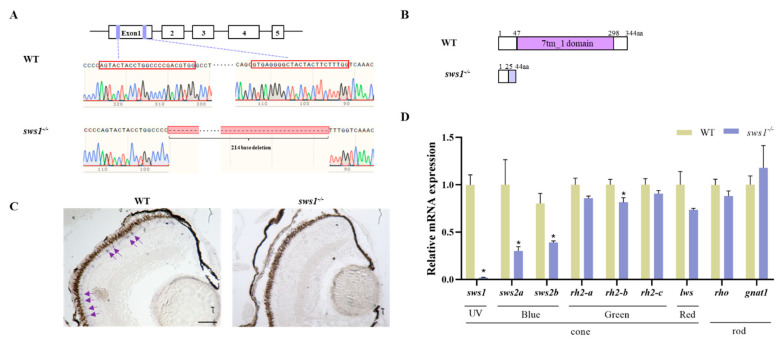
Establishment of *sws1*-knockout medaka. (**A**) *Sws1* gene and target sites of CRISPR/Cas9. *Sws1* consists of 5 exons (filled box). The sgRNA targets exon 1 and purple boxes indicate the sgRNAs targets, and red boxes indicate the sequences of sgRNA targets. DNA sequencing showing the *sws1* mutant lines (*sws1* ∆214). (**B**) Comparison of *sws1* ∆214 mutant with wild-type SWS1 protein structure. (**C**) In situ hybridization showing the expression patterns of *sws1* in WT and *sws1*^−/−^ medaka. Scale bar: 50 µm. (**D**) Relative mRNA expression of opsin genes in WT and *sws1*^−/−^ mutant medaka in the larval eyes at 6 dph. All data are expressed as means ± S.E.M (*n* = 6). * *p* < 0.05 per Student’s *t*-test.

We detected the expression pattern of *sws1* in medaka. *Sws1* was mainly expressed in the eye among adult tissues, and further RT-qPCR analysis showed that *sws1* expression was significantly higher in adult tissues than in larval tissues (Figure S1). Subsequently, we used the CRISPR/Cas9 technique to generate *sws1* mutant lines and better understand the function of *sws1* during medaka larval development. The *sws1^−/−^ larvae* line with 214 bp deletion (*sws1*^−/−^) in the 7-tm_1 domain resulted in frameshift and premature stop codon (Figure 1A,B). Compared with the WT, the *sws1*^−/−^ larvae showed a normal survival rate and morphology at 6 dph (Figure S2). In situ hybridization analysis revealed that *sws1* expression was significantly diminished in the *sws1*^−/−^ larvae (Figure 1C). The mRNA levels of *sws1* (*p* = 0.003), *sws2a* (*p* = 0.041), *sws2b* (*p* = 0.016), and *rh2*-*b* (*p* = 0.044) were down-regulated in the *sws1*^−/−^ mutant medaka at 6 dph (Figure 1D).

### 2.2. Decreased Food Intake in the Larvae of sws1 Knockout Medaka

**Figure 2:**
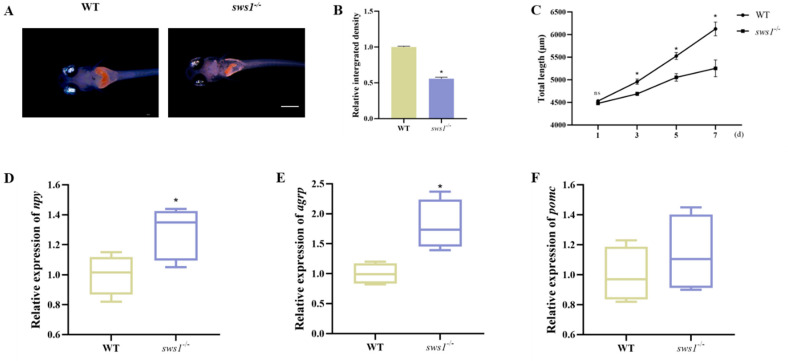
Analysis of WT and *sws1*^−/−^ medaka larval feeding. (**A**) Feeding assay of WT and *sws1*^−/−^ medaka using *Artemia* at 6 dph. Scale bar: 500 µm. (**B**) Quantification of the food intake between WT and *sws1*^−/−^ medaka larvae (*n* = 6/group). (**C**) Total length measurements of medaka within seven days of first feeding (*n* = 20/group); d, day. The transcriptional levels of orexigenic genes (*npy* (**D**) and *agrp* (**E**)) and anorexigenic gene (*pomc* (**F**)) in WT and *sws1*^−/−^ medaka larvae (*n* = 10/group, with 6 replicates). Results are shown as mean ± SEM. * *p* < 0.05. ns, not significant per Student’s *t*-test.

To assess feeding capacity in *sws1*^−/−^ medaka, we tested the food intake at 6 dph of the *sws1* mutant and WT. The results showed that there was significantly decreased food intake in the *sws1*^−/−^ medaka compared with the WT medaka at 6 dph (*p* < 0.001) (Figure 2A,B). Subsequently, we continued to analyze growth performance at the first-feeding stage. There was reduced growth compared with the WT within seven days of first feeding (*p* < 0.05) (Figure 2C). The relative expression levels of orexigenic genes (*npy* (*p* = 0.034) and *agrp* (*p* = 0.012)) in *sws1*^−/−^ mutant larvae were significantly increased after feeding (Figure 2D,E). No difference in the mRNA levels of the anorexigenic gene (*pomc*) was observed between the WT and *sws1*^−/−^ medaka (*p* > 0.05) (Figure 2F).

### 2.3. sws1 Deficiency in Larvae Affected Retinal Lamination and Reduced Expression of Phototransduction Genes

**Figure 3:**
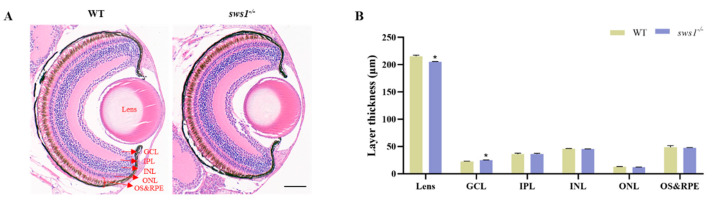
Histological analysis of the retina in larval medaka at 6 dph. (**A**) Histological morphology of the retina with H&E staining. Scale bar: 40 µm. (**B**) The thickness of lens, GCL, IPL, INL, ONL, OS, and RPE (*n* = 3). GCL, ganglion cell layer; IPL, inner plexiform layer; INL, inner nuclear layer; ONL, outer nuclear layer; OS&RPE, outer segment and retinal pigment epithelium. Error bars represent SEM, and asterisks indicate * *p* < 0.05 per Student’s *t*-test.

**Figure 4:**
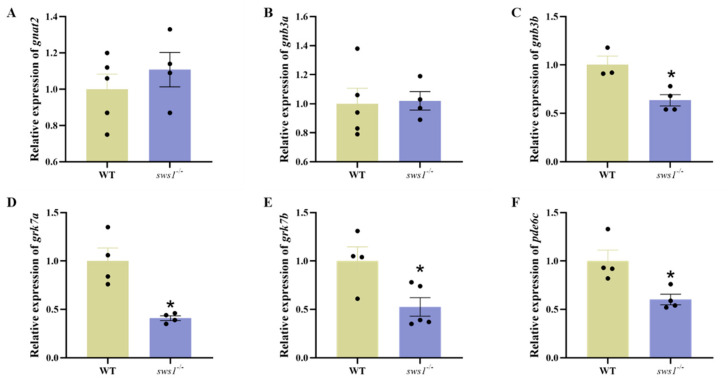
Relative mRNA levels of phototransduction-related genes in the larval eyes at 6 dph medaka larvae. (**A**) *gnat2*. (**B**) *gnb3a*. (**C**) *gnb3b*. (**D**) *grk7a*. (**E**) *grk7b*. (**F**) *pde6c*. Relative expression levels are presented as mean ± SEM (*n* = 6). * *p* < 0.05 per Student’s *t*-test.

In order to characterize the retina in medaka *sws1*^−/−^, we performed histological analysis using hematoxylin–eosin (H&E) staining. The thickness of the lens in the *sws1*^−/−^ larvae was significantly reduced (*p* = 0.018), whereas the thickness of the ganglion cell layer (GCL) was significantly increased (*p* < 0.023). The results showed no significant change in the thickness of the inner plexiform layer (IPL), inner nuclear layer (INL), outer nuclear layer (ONL), outer segment (OS), and retinal pigment epithelium (RPE) between the WT and *sws1*^−/−^ mutant larvae (*p* > 0.05) (Figure 3). In addition, we found that the transcript levels of genes involved in phototransduction (*gnb3b* (*p* = 0.015), *grk7a* (*p* = 0.019), *grk7b* (*p* = 0.025), and *pde6c* (*p* = 0.019)) also decreased in the *sws1*^−/−^ mutant larvae (Figure 4C–F).

### 2.4. Disruption of sws1-Impaired Medaka Larvae Swimming Behavior

**Figure 5:**
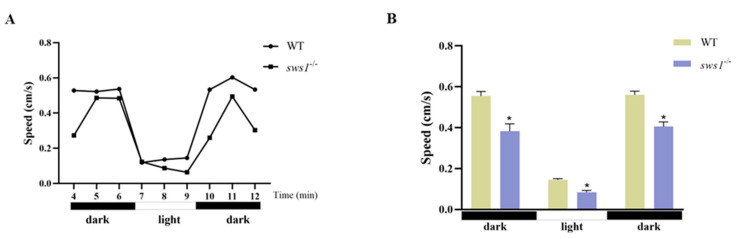
Changes in swimming behavior in light/dark behavioral tests. Behavioral traces (**A**) and swimming speed (**B**) of WT and *sws1*^−/−^ medaka at 6 dph (*n* = 12/group). Error bars represent SEM, and asterisks indicate * *p* < 0.05 per Student’s *t*-test.

Next, we performed a photoperiod stimulation test at 6 dph for WT and *sws1*^−/−^ mutant larvae. Compared with the WT, the *sws1*^−/−^ larvae displayed a decrease in swimming speed during periods of dark-light and light-dark transition stimulation (*p* < 0.05) (Figure 5).

### 2.5. Induced Oxidative Stress in sws1-Deficient Medaka Larvae

**Figure 6:**
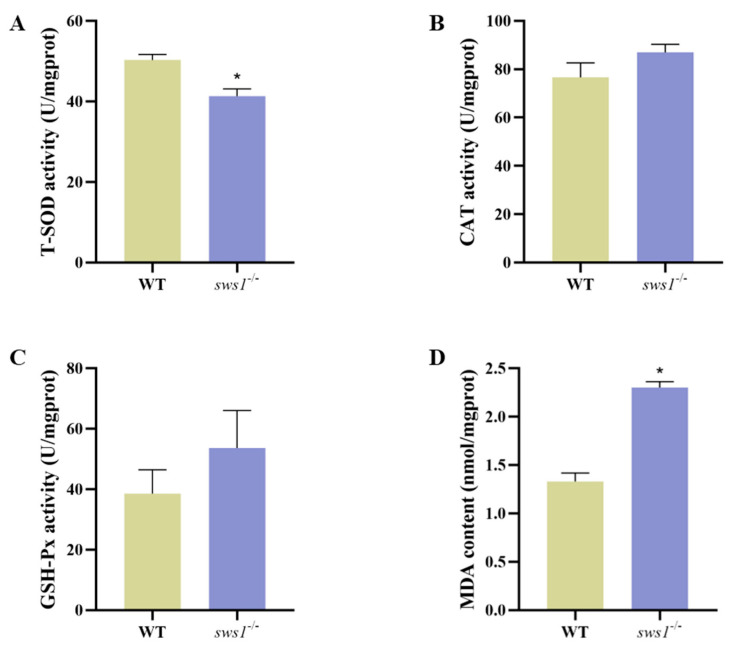
Antioxidant indices in *sws1* knockout medaka larvae. (**A**) Total superoxide dismutase (T-SOD) activity. (**B**) Catalase (CAT) activity. (**C**) Glutathione peroxidase (GSH-Px) activity. (**D**) Malondialdehyde (MDA) content. Error bars, mean ± SEM. * *p* < 0.05 per Student’s *t*-test.

To evaluate the oxidative stress activities in medaka larvae, the activities of total superoxide dismutase (T-SOD), catalase (CAT), glutathione peroxidase (GSH-Px), and malondialdehyde (MDA) were detected. Compared with the WT larvae, CAT activity and GSH-Px content were not significantly changed, but the T-SOD activity was significantly reduced (*p* = 0.007) (Figure 6A–C). Meanwhile, we observed an MDA content in the *sws1*^−/−^ mutant larvae that was dramatically increased (*p* < 0.001) (Figure 6D).

### 2.6. Depletion of sws1 Led to Retinal Cell Apoptosis in Medaka Larvae

**Figure 7:**
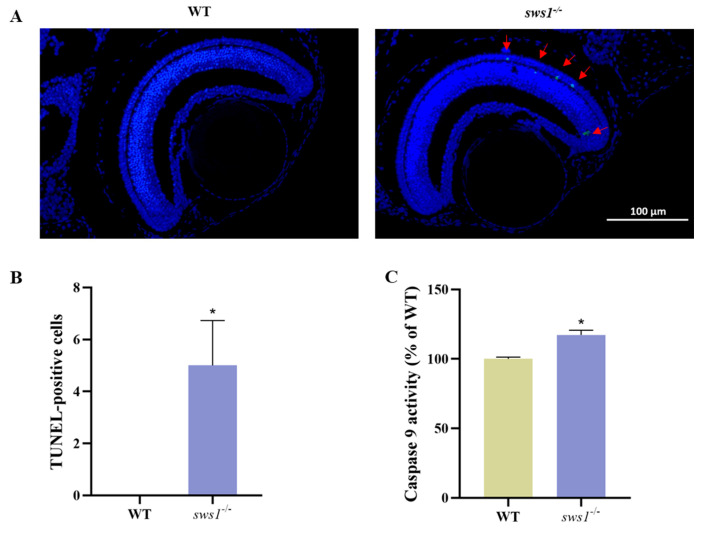
Comparison of retinal cell apoptosis in WT and *sws1*^−/−^ medaka larvae. (**A**) TUNEL-positive cells in WT and *sws1*^−/−^ medaka at 6 dph. The red arrows indicate the TUNEL- positive signal. Scale bar: 100 µm. (**B**) The number of TUNEL-positive cells in the retina (*n* = 3). (**C**) Caspase-9 activity in 6 dph medaka larvae (*n* = 100/group, with 6 replicates). The values shown are means ± SEM. * *p* < 0.05 per Student’s *t*-test.

To investigate whether apoptosis activation mediates the effects of visual function in medaka larvae, apoptosis was assessed in the WT and *sws1*^−/−^ larvae at 6 dph. There were few TUNEL-positive cells in the WT retinas. In comparison, the *sws1*^−/−^ mutant larvae had significantly more apoptotic cells in the eye (*p* = 0.045) (Figure 7A,B). Caspase-9 is a relatively upstream caspase in apoptotic signaling [20]. At 6 dph, the activity of caspase-9 in the *sws1*^−/−^ medaka larvae was found to be higher than that in the WT larvae (*p* = 0.005 (Figure 7C).

## 3. Discussion

In this study, we created a medaka mutant line with a 214 bp deletion via CRISPR/Cas9 gene editing technology. The deletion caused the loss of seven transmembrane domain 1 (7-tm_1). We observed that the knockout of *sws1* caused impaired feeding ability and hypoactivity in the first feeding stage. Histological analysis and subsequent experiments indicated that impaired feeding ability and hypoactivity might be the causes of the affected retinal structures and downregulation of phototransduction related genes. We further found that *sws1*^−/−^ individuals had reduced resistance to oxidative stress and increased apoptosis in the larval eye.

Visual perception is the main sensory way for fish larvae to detect and capture prey [21]. Fish with photopic vision utilize more cones, whereas those with scotopic vision, or dark-adapted eyes (twilight vision), use rods [22]. Ultraviolet vision plays a crucial role in identifying zooplankton because UV light scatters in water bodies, while near-transparent zooplankton absorb UV light in contrast [23,24]. The UV-sensitive opsin (*sws1*) expressed by UV cones is sensitive to UV light, and its expression level determines the sensitivity of UV cones [25]. Flamarique [9] treated young rainbow trout with thyroid hormone, resulting in the reduction in single cone cells expressing *sws1* mRNA in the retina and the reduction in sensitivity to zooplankton. Similarly, the *tbx2b* zebrafish mutant with reduced UV cones also found zooplankton with a smaller mean positional distance and angle than wild zebrafish [26]. Yoshimatsu et al. [27] further revealed that the UV cone is the main input of the visual predator–prey circuit of zebrafish larvae through two-photon imaging in vivo, transcriptome, and computational models. In *sws1* knockout medaka, food intake was shown to be significantly reduced, accompanied by slow growth during the first feeding phase (Figure 2A–C). Correspondingly, the appetite–orexigenic genes (*npy* and *agrp*) were significantly upregulated after feeding in the *sws1* mutants (Figure 2D,E). Our explanation is that the *sws1*^−/−^ mutant larvae have an impaired feeding ability and still do not reach satiation after half an hour of feeding compared with the WT, so their appetitive genes are still upregulated. Numerous studies have implicated *npy* and *agrp* in the regulation of appetite and food intake in teleosts [28,29,30]. In mice, the NPY^+^ and AgRP^+^ signals in the hypothalamic arcuate nucleus (ARC) in the fasting group were significantly stronger than those in the feeding group [31]. Our result is consistent with the expression of *npy* and *agrp* during fasting. The mediating and integrating role of *sws1* in feeding and appetite needs to be investigated in further studies.

The retina is a highly complex tissue structurally composed of three layers of nerve cell bodies (ONL, INL, and GCL) and two layers of synapses (OPL and IPL) [32]. In each layer, there are specific cells with varied physiological functions. An important retinal neuron is the GCL, which connects the retina to the optic tectum in the brain [33]. Histological results showed that the thickness of the GCL was significantly increased, whereas the lens became thinner in the *sws1*^−/−^ mutant larvae (Figure 3), which may have led to impaired visual function [34]. The spectral specificity of visual pigments is determined via the opsin synthesized by cones and rods in the ONL [35]. Here, there was no obvious histological damage in the ONL, indicating that it is likely a molecular effect. Our quantification showed that the transcription levels of *sws1*, *sws2a*, *sws2b,* and *rh2*-*b* were significantly decreased in *sws1*^−/−^ mutant larvae (Figure 1D). Opsin accumulation in photoreceptors may be reduced as transcription levels of opsin genes decline [36]. In addition, many previous studies have suggested that photoreceptor defects may lead to the decreased sensitivity of fish larvae to light stimulation [37,38,39].

Subsequently, we tested the sensitivity of the fish larvae to light stimulation, and we discovered that the *sws1*^−/−^ larvae showed a reduction in swimming speed compared with the WT larvae in the light/dark behavior test (Figure 5). The light/dark cycle is considered to be the main factor that entrains the biological rhythm of teleosts through its influence on feeding physiology and gene expression [40,41,42]. Studies have shown that both clock genes and feeding behavior exhibit a circadian pattern [43]. Rods, cones, and melanopsin are the only photoreceptors that contribute to circadian light entrainment [44]. Therefore, *sws1* loss may affect circadian photoentrainment. In addition, intrinsic photosensitive retinal ganglion cells (ipRGCs) are also involved in regulating circadian photoentrainment, whereas ipRGCs reside in the GCL [45,46]. In this study, changes in the thickness of ganglion cells may also have affected circadian photoentrainment. Furthermore, the genes *gnb3b*, *grk7a*, *grk7b,* and *pde6c*, which are associated with phototransduction, were downregulated in *sws1*^−/−^ mutant medaka at 6 dph (Figure 4). In phototransduction, opsin converts optical signals into electrical signals by generating electrochemical signals from captured photons via pigments and intracellular mechanisms [2,47]. *Gnb3b*, *grk7a*, *grk7b,* and *pde6c* form the downstream phototransduction pathway of cone opsin [48,49]. In zebrafish, *gnb3b* disruption exhibited aberrant cone photoreceptor formation [50]. It was also found that *grk7a*-knockdown larvae had slower photoresponse recovery and that their temporal contrast sensitivity was reduced [51]. Also, similar results were observed in *pde6c* zebrafish mutant larvae [52]. In addition, it has been reported that the thinning of the GCL prevents electrical signals from being transmitted to the central nervous system [53,54]. Therefore, the decreased activity of the *sws1*^−/−^ mutant larvae may have mainly been due to impaired retinal light signal reception and transmission.

Our study further found that the absence of *sws1* reduced the resistance of larvae to oxidative stress. The levels of CAT and GSH-Px in the WT and *sws1*^−/−^mutant larvae were not statistically significantly different (Figure 6B,C). These results suggested that CAT and GSH-Px may not be involved in antioxidation to *sws1* in medaka larvae. The T-SOD activity and MDA content in the *sws1*^−/−^ mutant larvae were significantly altered (Figure 6A,D), demonstrating that *sws1* disruption caused oxidative damage. Excess free radicals lead to the overproduction of MDA, a lipid peroxidation product that indicates the severity of oxidative stress [55]. On the other hand, oxidative stress is one potential factor in the cellular apoptosis process. Oxidative stress releases mitochondrial cytochrome c, which activates apoptogenic proteins [56,57]. The loss of *sws1* led to retinal cell apoptosis in medaka larvae (Figure 7A,B). Additionally, caspase (cysteine-requiring aspartate protein) is a protease family that plays an important role in the process of cell apoptosis, and caspase-9 is the initiator of apoptosis signal transduction [58,59,60]. In our study, the activity of caspase-9 in the *sws1*^−/−^ medaka larvae was significantly increased compared with the wild-type medaka (Figure 7C), suggesting that the deletion of *sws1* resulted in the activation of caspase-9. Our characterization of retinal cell apoptosis in *sws1* genes is similar to that of Blanch et al. [61]. Moreover, previous studies have shown that eye development defects in zebrafish larvae are caused by the apoptosis of neuronal progenitor cells in the eye [62]. Therefore, we speculated that the abnormal development of the eye in the *sws1*^−/−^ mutant larvae is also related to the increased level of apoptosis.

In summary, we revealed an essential role of *sws1* in vision-guided behavioral alteration and visual development. Our results implicate *sws1* signaling as a regulator of reactions to prey capture and light–dark transitions in larval medaka, with *sws1*^−/−^ medaka larvae showing decreased food intake and swimming speed during the period of dark–light and light–dark transition stimulation. As another new discovery, we observed microphthalmia and increased retinal cell apoptosis in *sws1* knockout medaka larvae, which may have been related to oxidative stress. Our results provide insights into understanding the mechanisms of *sws1* mutation-mediated vision-guided behavioral and visual developmental alteration in medaka.

## 4. Materials and Methods

### 4.1. Medaka Lines and Maintenance

The wild-type medaka is an orange strain, which was kept at 26–28 °C and a 14 h light/10 h dark cycle. Medaka embryos were raised at 28 °C in medaka embryo medium (MEM) [63]. Larvae at 6 days post-hatching (dph) were given live *Artemia* twice daily after the yolk sac had almost been consumed. All medaka protocols were approved by the Institutional Animal Care and Use Ethics Committee of Huazhong Agricultural University (approval reference number HZAUFI-2020-0024).

### 4.2. Generating sws1^−/−^ Mutants Using CRISPR/Cas9 Technology

The single-guide RNAs (sgRNAs) of medaka *sws1* (ENSORLG00000019293) gene were designed using CCTOP web (https://cctop.cos.uni-heidelberg.de/). The sequences of single-guide RNAs (sgRNAs) and PCR primers are shown in Supplementary Table S1. sgRNAs were cloned into pMD-18T vector and were synthesized using TranscriptAid T7 High Yield Transcription kit (Thermo, Scientific, San Diego, CA, USA). The compounds of sgRNAs (50 ng/µL) and Cas9 protein (NEB, Ipswich, MA, USA) were coinjected into one-or two-cell stage wild-type embryos. The F0 medaka were outcrossed with the wild type to generate F1 medaka. The target region was amplified using ordinary PCR with *sws1* test-F and *sws1* test-R. The reaction conditions were 95 °C for 30 s, 60 °C for 30 s, and 72 °C for 30 s for 40 cycles. We detected that the *sws1* mutation type is a large deletion, and we could preliminarily judge whether the F1 generation was a heterozygous mutant using PCR. Then, the mutation type was determined via sequencing. The F1 heterozygous with the same mutation in-crossed to obtain F2 homozygous, and all experiments were conducted with F3 homozygous.

### 4.3. Histological Assessment, In Situ Hybridization (ISH), and TUNEL Staining

Whole medaka at 6 dph were preserved in 4% paraformaldehyde solution (PFA) for 24 h, dehydrated in 70–100% ethanol, embedded in paraffin, and sectioned in thicknesses of 4 μm (Leica, Wetzlar, Germany). Then, the sections were stained with (H&E) according to standard protocols. The slides were imaged via slice digital scanning (Pannoramic250, Pannoramic250 MIDI, 3D HISTECH). The thickness of each retinal layer in right eye was measured using ImageJ 1 software according to the Chen et al. method [37].

For in situ hybridization (ISH), the 6 dph sections hybridized with the DIG-labeled RNA probes (Roche, Basel, Switzerland) at 65 °C for 12–16 h and the hybridization signals were visualized with nitroblue etrazolium chloride (NBT)/5-bromo-4-chloro-3-indolyl phosphate (BCIP) (NBT/BCIP) (Sigma, Cibolo, TX, USA) staining, as described in our previous study [64]. The signals from the right eye of the larvae were observed under an optical microscope (Olympus, Tokyo, Japan). The probes primers are listed in Supplementary Table S1.

A TUNEL staining kit (catalog no. G1501) (Servicebio, Wuhan, China) was used to identify apoptotic cells in the medaka 6 dph sections, following the manufacturer’s instructions. The TUNEL-positive cells on the slides were observed using a fluorescence microscope (NIKON ECLIPSE C1, Tokyo, Japan), and the quantification of TUNEL-positive cells was performed by manually counting the number of positively labeled cones in the retina.

### 4.4. Light/Dark Behavior Analysis

Light/dark behavioral tests were conducted between 15:00 and 17:00 using a DanioVision Observation Chamber (Noldus Information Technology, Wageningen, The Netherlands) linked to Etho Vision XT13 software. The 6 dph larvae were plated into a 24-well plate (diameter 1.56 cm wells) with 1 mL of MEM (individual larvae per 24-well plate). The larvae were acclimated for 10 min at 28 °C, and the larval locomotor activity was tested in response to dark–light conversion (3 min light/3 min dark/3 min light/3 min dark) based on the protocol by Huang et al. [65] with some modifications. The average swimming speed (cm/s) for each individual larva was collected every 60 s, and each experiment was repeated 3 times. Further analysis was performed using custom Open Office Org 2.4 software.

### 4.5. Larvae Feeding Assays

For larvae food intake, 6 dph larvae were fed with *Artemia* in wells of a 6-well plate with 8 mL of MEM (diameter 3.48 cm wells) for 30 min (6 larvae per 6-well plate). Then, larvae were anesthetized with MS-222 and some larvae were fixed with 4% PFA overnight for food intake analysis, and others were frozen with liquid nitrogen for quantitative analysis of appetite genes. The orange area of *Artemia* in the digestive tract was photographed using a stereomicroscope and measured with Image J 1 software. The amount of food ingested by medaka larvae was determined following the procedure described previously [66].

### 4.6. Growth Performance and Survival Rate

For the growth performance and survival rate, 6 dph larvae were fed with abundant *Artemia* twice daily for 7 days. Twenty WT and *sws1*^−/−^ medaka larvae were randomly selected, anaesthetized, and fixed with 4% PFA for total-length measurement using Image J1. The experiment was repeated 3 times.

### 4.7. Biochemical Analyses and Caspase Activity Assay

The homogenate (20%) of larvae (*n* = 100/group) from each replicate was centrifuged at 8000 rpm at 4 °C for 10 min, and the supernatant was collected for subsequent analysis. The total superoxide dismutase (T-SOD) activity, catalase (CAT) activity, glutathione peroxidase (GSH-Px) content, and malondialdehyde (MDA) content were measured with commercially available kits (T-SOD assay kit (catalog no. A001-1-2), CAT assay kit (catalog no. A007-1-1), GSH-Px assay kit (catalog no. A005-1-2), and MDA assay kit (catalog no. A003-1-2)) purchased from Jian-cheng Institute of Biotechnology (Nanjing, China) and the caspase-9 activity was detected with a Caspase-9 Activity Assay Kit (catalog no. C1157) (Beyotime, Haimen, China) according to the manufacturer’s instructions.

### 4.8. RNA Isolation and Quantitative RT-PCR

All fish were sampled in the light phase of the light/dark cycle. The adult fish tissues (*n* = 3 individuals) and 6 dph larval eyes (*n* = 6 individuals) were collected and frozen in liquid nitrogen. Total RNA was extracted from each sample according to the RNAiso instructions and the cDNA reverse-transcribed using a HiScript^®^ III 1st Strand cDNA Synthesis Kit (Vazyme, Nanjing, China). Quantitative RT-PCR was performed with ChamQ universal SYBR qRT-PCR master mix (Vazyme, Nanjing, China) on a CFX 96 Touch System (Bio-Rad, Hercules, CA, USA). The data were collected using the 2^−ΔΔCt^ value method and normalized to *β*-*actin* [67]. The primers of opsin genes and phototransduction-related genes are set in Table S2, and the primers of appetite genes were carried out as described previously [68].

### 4.9. Statistical Analysis

All results are presented as means ± standard error of the mean (S.E.M), and the normality of the data was first tested using the Shapiro–Wilk test. The differences were analyzed using Student’s *t*-test, and differences were considered significant at *p* < 0.05. All statistical analyses were carried out using IBM SPSS Statistics 25 software.

## Data Availability

All data are available from the corresponding author by request.

## Supplementary Materials

The following supporting information can be downloaded at: https://www.mdpi.com/article/10.3390/cells12172157/s1, Figure S1: The expression pattern of *sws1* in medaka; Figure S2: The survival and morphology of WT and *sws1*^−/−^ medaka larvae; Table S1: The primers used for CRISPR/Cas9; Table S2: The primers for qRT-PCR.
