# Supplementary material for: Altered Visual Function in Short-Wave-Sensitive 1 (sws1) Gene Knockout Japanese Medaka (Oryzias latipes) Larvae
Source: Cells. 2023 Aug 28;12(17):2157. doi: 10.3390/cells12172157 (PMC10486665; doi:10.3390/cells12172157)
Supplement: Supplementary file 1 [file cells-12-02157-s001.zip › cells-2487438-supplementary.pdf]

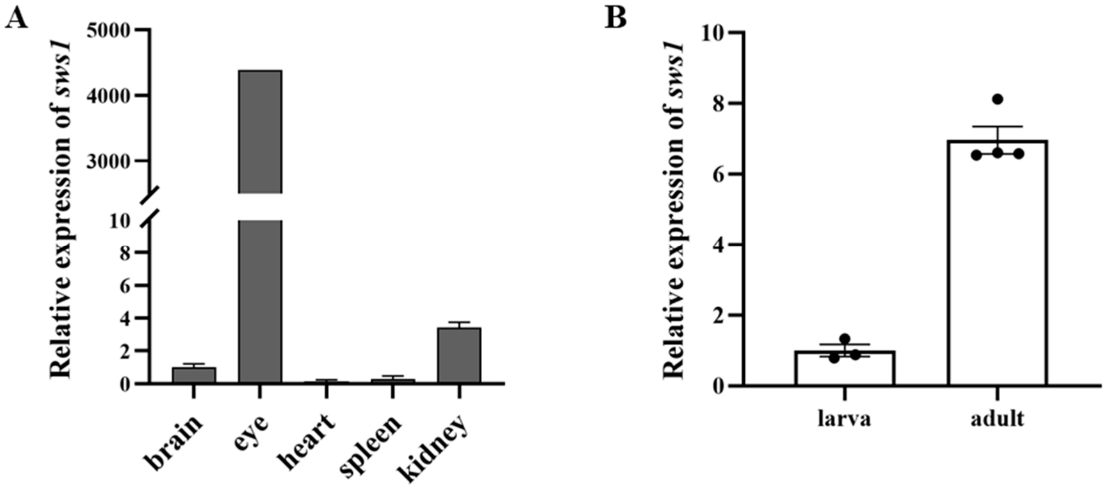

Figure S1 The expression pattern of *sws1* in medaka. (A) Detection of *sws1* mRNA expression in different tissues by RT-PCR (n = 3). (B) The mRNA level of *sws1* in larval and adult medaka (n = 6). All data were expressed as means ± S.E.M.

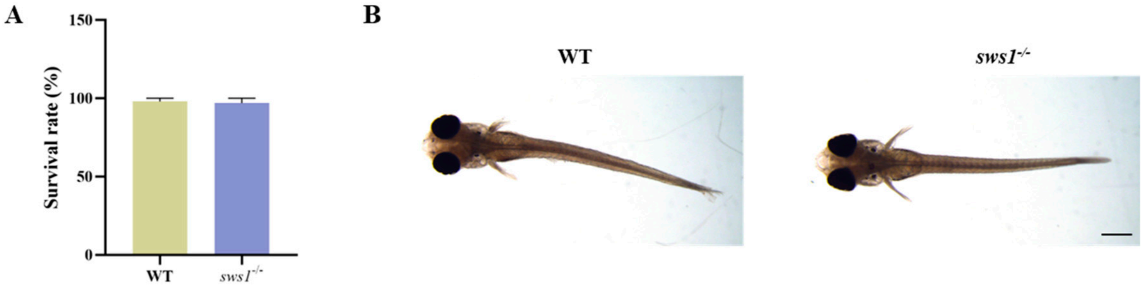

Figure S2 The survival and morphology of WT and *sws1*$^{-/-}$ medaka larvae. (A) Statistic analysis of survival rate in WT and *sws1*$^{-/-}$ medaka larvae. (B) Photographs of larvae at 6 dph for WT and *sws1*$^{-/-}$ medaka. Scale bar: 500 μm.

Table S1 The primers used for CRISPR/Cas9.

Gene	Sequence (5'-3')
sws1 guide RNA1	GTACTACCTGGCCCCGACGTGGG
sws1 guide RNA2	GTGAGGGGCTACTACTTCTTTGG
sws1 test-F	TCAGCTTCACGATGGGAAAAT
sws1 test-R	CAGGCCTGCAGAGAAAGCAC
sws1 ISH-F	TGGAGCCTTCAAGTTTGGCA
sws1 ISH-R	TGACGAGCCGGTAGTCTTTG

Table S2 The primers for *q*RT-PCR.

Gene	Gene No.	Forward (5'-3')	Reverse (5'-3')
actin	NM_001104808.1	TATCATTCGCCTGAAACCGAT	CTTTGCACATGCCAGATCCG
sws1	NM_001104656.1	ACAAAGACTACCGGCTCGTCA	GCTTCCTCCATCTTCTTTCCGA
sws2a	XM_004069227.4	TGTTACCTGGGTGTGTGCTC	GAACCGCGAAGCAGAAACAG
sws2b	NM_001104654.1	TTCTGCTTTGCCGTCCCTT	AAGCAACAACCATGACGACCA
rh2-a	NM_001104655.1	TTTGGTTGGCTGGTCAAGGT	CACAAGGCTGCCATAGGTGA
rh2-b	XM_004086130.4	TGCACTTCTTCGTCCCAGTC	CCAAGCTACCAGGAAGCCAA
rh2-c	XM_004086132.4	CTTCTGTGCCGTTGAGGGAT	ACAAGCCAGAGCCATTACCC
lws	XM_004069094.4	TGAGGGCTATGTGGTCTCCA	ACCTGCTCCATCCAAAGACG
rho	NM_001104695.1	CTCGTTACATCCCAGAGGGC	ATGATGACAACCATGCGGGT
gnat1	XM_004084384.4	AAGCCCATCTGAGCATGTGT	TGATGATGTCGGTTACGGCA
gnat2	XM_004070401.3	GTGAGTCGGGAAAAAGCACC	CCAGAGCCAGAACCGAAGTC
gnb3a	XM_004077988.4	CAGCCTCAAAGACCAGATTACGG	TCATCTGCACACGTCCCAC
gnb3b	NM_001104851.1	GAAAGCTGAAATGGATGCACTGAA	ACATGCTCCCATCATTTGCTG
grk7a	NM_001104661.1	CCACCAAGGACGTCATCCA	CGTGTTTCCTCGGATCCTC
grk7b	XM_004075815.3	TGAGAGGCAGAAGATTACTGACA	GCACAACACAAACCTCACCAAA
pde6c	XM_004080270.3	CGGCAAACTCCAAAAAGCCA	TGAGGGGGAAGGTGAGCATA
